# Climate and Dispersal Ability Limit Future Habitats for Gila Monsters in the Mojave Desert

**DOI:** 10.1002/ece3.71008

**Published:** 2025-03-17

**Authors:** Steven J. Hromada, Jason L. Jones, Jocelyn B. Stalker, Dustin A. Wood, Amy G. Vandergast, C. Richard Tracy, C. M. Gienger, Kenneth E. Nussear

**Affiliations:** ^1^ Program in Ecology, Evolution and Conservation Biology, Department of Geography University of Nevada Reno Nevada USA; ^2^ Conservation Science Partners, Inc Truckee California USA; ^3^ Current Address: Fresno Chaffee Zoo Fresno California USA; ^4^ Nevada Department of Wildlife Las Vegas Nevada USA; ^5^ WEST, Inc Las Vegas Nevada USA; ^6^ Center of Excellence for Field Biology, Department of Biology Austin Peay State University Clarksville Tennessee USA; ^7^ United States Geological Survey San Diego California USA; ^8^ Department of Biology University of Nevada Reno Nevada USA

**Keywords:** climate change, ensemble species distribution model, movement selection, reptile

## Abstract

Describing future habitat for sensitive species can be helpful in planning conservation efforts to ensure species persistence under new climatic conditions. The Gila monster ( *Heloderma suspectum* ) is an iconic lizard of the southwestern United States. The northernmost range of Gila monsters is the Mojave Desert, an area experiencing rapid human population growth and urban sprawl. To understand current and potential future habitat for Gila monsters in the Mojave Desert, we fit ensemble species distribution models using known locations and current environmental variables known to be important to the species' biology. We then projected future suitable habitat under different climate forecasts based on IPCC emission scenarios. To ensure that Gila monsters would be able to disperse to newly suitable habitat, we fit Brownian Bridge movement models using telemetry data from two locations in Nevada. This model indicated that Gila monsters prefer to move through areas with a moderate slope and higher shrub cover. Modeled current suitable habitat for Gila monsters in Nevada was primarily in rugged bajadas and lower elevations at the bases of mountain ranges. Predictions of potential future habitat suggested that overall habitat suitability through 2082 would remain relatively stable throughout the study area in the lower emissions scenario, but in the high emissions scenario potential habitat is greatly reduced in many lower‐elevation areas. Future habitat areas at higher elevations under the high emissions scenario showed moderate increases in suitability, though occupancy would likely be limited by Gila monster dispersal capabilities. Finally, we determined how well the protected area network of our study area encompassed future Gila monster habitat to highlight potential opportunities to protect this important species.

## Graphical abstract

We assessed how suitable habitat for Gila monsters will change under climate change scenarios, limiting future habitat to areas that Gila monsters can potentially disperse to. We find that without this limitation, future Gila monster habitat will be overestimated.

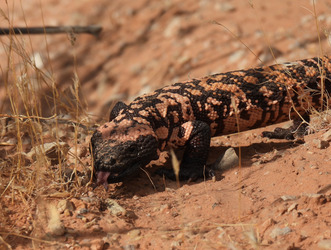

## 1 Introduction

Alterations of the earth's climate will certainly affect the distribution of species, which will have impacts on ecosystem processes, species extinction, and human society (Pecl et al. 2017; Thomas et al. 2004; Thuiller 2007). As global temperatures increase, species respond by shifting their geographic range to match their environmental tolerances and exploit new opportunities (Chen et al. 2011; Lenoir and Svenning 2015). Predicting potential future distributions of suitable habitat for species can be important in planning conservation measures, such as reserve placement and protected corridors between them, that can preserve species' habitat and habitat connectivity under changing climatic conditions (Anderson 2013; Elith and Leathwick 2009; Guisan et al. 2013). Correlative species distribution models, which relate known locations of a species to environmental conditions, provide a method that allows for quantification of current suitable habitat (Franklin 2009). These models can then be projected using forecasts of future climatic conditions to predict where suitable habitat may exist under new environmental conditions (Guisan and Zimmermann 2000). Species distributions can be modeled using a variety of algorithms and combining the outputs of different algorithms using an ensemble modeling approach can be a powerful method to increase the robustness of predictions (Araújo and New 2006; Thuiller et al. 2009).

Although an area may become newly suitable for a species, it may not be available due to restrictions in species movement abilities or other aspects of the species' biology (Briscoe et al. 2019; Inman et al. 2022). One major barrier to the occupation of future suitable habitat is the dispersal capabilities of a species. If a species is unable to disperse into areas that become suitable under new conditions, those areas will likely remain unoccupied. Without accounting for this potential limitation, predicted habitat under future conditions may be larger in area than the area that can be occupied by the species, resulting in an underestimation of the threat of environmental change. Dispersal limitation is likely a factor for many species of reptiles that will limit the occupancy of future habitat and lead to declines in the area and extent of species distributions due to their small body size, low vagility, and physiological constraints (Araújo et al. 2006; Bestion et al. 2015; Clobert 2012; R. D. Inman et al. 2022). Accounting for this dispersal limitation in forecast models will provide a more realistic estimation of future habitat that can be potentially occupied by a species.

**FIGURE 1:**
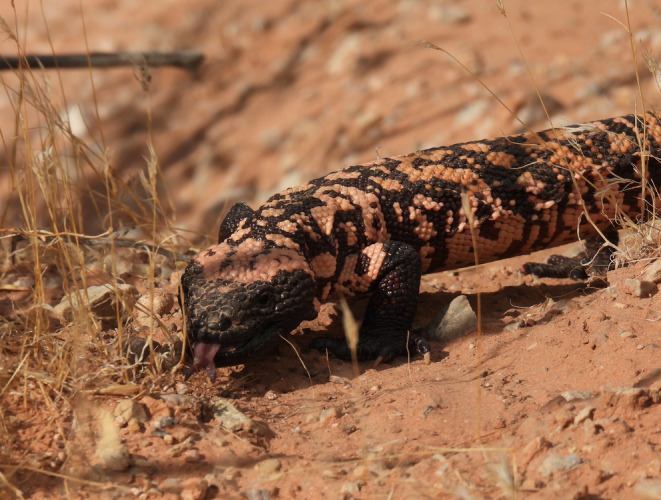
Female Gila monster ( *Heloderma suspectum* ) used in telemetry study at Site C, Clark County, NV.

The Gila monster ( *Heloderma suspectum* ) is an iconic lizard of the southwestern deserts of the U.S. and northern Mexico that reaches the northern extent of its geographic range in the Mojave Desert of Nevada, Utah, California, and Arizona (Figure 1; Beck 2005). There has long been concern over the conservation of this unique species, and how human activities may influence populations, especially as human development in the southwestern United States continues to grow and modify desert habitats (Bogert and Martín del Campo 1956; Hughson 2009). The species is listed as “Near Threatened” by the International Union for the Conservation of Nature (IUCN), and is protected from collection across its range (Hammerson et al. 2007). Habitat requirements and the distribution of the species within the Mojave Desert remain poorly understood despite considerable interest; previous work suggests that geology and shelter site availability are crucial aspects for determining the suitability of habitat for this species (Beck and Jennings 2003; Gienger 2003). Understanding the needs of this ecologically unique species is important in informing the management of public lands alongside continuing land use changes within desert landscapes.

Here, we present a model of predicted Gila monster habitat in the Mojave Desert and predict how suitable habitat may change under future climate scenarios using an ensemble modeling framework (Araújo and New 2006). We expected that current and future habitat suitability for Gila monsters would be limited by climatic and other abiotic variables that determine appropriate foraging opportunities and shelter locations. We anticipated that future habitat would not be accessible to the species due to limited dispersal capacity; to account for these limits we restricted newly suitable available habitat by modeling Gila monster movement with telemetry data from two areas in Clark County, Nevada. We demonstrate that suitable habitat will likely differ depending on climate forecasts, and that many areas that are projected to be newly suitable for the species may not be accessible via dispersal. Finally, we perform a Gap Analysis on the suitable habitat for Gila monsters in both present and under future conditions to assess how much habitat is covered by the protected areas network of the region, with the expectation that protected lands in higher elevations may serve as future refugia for the species.

## 2 Methods

### 2.1 Habitat Suitability Model

We modeled currently suitable habitat for Gila monsters in an area within the northeastern Mojave Desert. This area encompassed all known localities for the species within Nevada and Utah, most of the localities from California (excluding the disjunct records in the Chocolate Mountains of the Colorado Desert), and localities from Mohave County, Arizona.

We used an ensemble modeling approach that incorporated four different algorithms: Random Forests (RF; R package randomForest v4.7–1.1, (Liaw and Wiener 2002)), Generalized Boosted Models (GBM; gbm package v2.1.8, (Greenwell et al. 2019)), Generalized Additive Models (GAM; mgcv package v1.8–39 (Wood 2001)), and MaxEnt (Phillips and Dudík 2008). The use of multi‐algorithm ensemble models renders predictions less susceptible to biases, assumptions, or limitations of any individual algorithm, while broadening the types of environmental response functions that can be identified (Araújo and New 2006). Moreover, empirical evaluations have found GAM, RF, and MaxEnt to be consistently strong performers among habitat distribution modeling algorithms (Franklin 2009). All habitat modeling was conducted in R version 4.1.3 (R Core Team 2018).

Gila monster presence data were obtained from several sources, including published articles and reports (Nussear et al. 2011; Southwest Ecology LLC 2018), online databases (iNaturalist, GBIF, Vertnet, and Herpmapper), and other research efforts by county and state government agencies. We verified that records representing photo vouchers were Gila monsters. We also digitized the points representing known localities for the species in California from Thomson 2016. Collectively, this resulted in 3879 occurrences. Because presence points were spatially aggregated, which can lead to substantial bias in model predictions, we first rasterized the presence points to the modeling resolution (i.e., such that only one presence point could occur within each 250 m modeling grid cell). We subsequently applied a geographically stratified resampling procedure in which a maximum of four observations could be sampled from cells on a uniform grid at a larger spatial resolution than the modeling extent (1000 m). This spatial thinning of presence points can be effective at reducing spatial bias under a variety of conditions (Fourcade et al. 2014), which we confirmed with variograms of habitat parameters for differing thinning numbers and grid sizes. The number of occurrences used for modeling after thinning consisted of 756 localities.

True absence points were not available for this species. For this reason, all models were fit using background points (pseudo‐absences) generated using the Bioclim climate envelope model in the dismo package (v1.3–5 [Hijmans et al. 2022b]), with a threshold of 0.05 with an equal number of absence and presence points. This method of pseudo‐absence selection uses a basic envelope model and selects points outside of the general envelope. The MaxEnt algorithm uses an internally generated set of 10,000 random background points (Phillips et al. 2006), while the GAM, GBM, and RF models were fit with an equal number of presence and background points (Barbet‐Massin et al. 2012).

### 2.2 Environmental Covariates for Habitat Suitability Model

We used a suite of environmental layers that are related to Gila monster ecology, and that we expected to play a role in determining the distribution of the species. These included topographic, geologic, and climatic variables. Descriptions, original resolution, source, and reasons for inclusion of each covariate are provided in Table 1. All covariates were resampled to a common 250 m resolution using bilinear interpolation (raster package v3.6–11 for R [Hijmans et al. 2022a]). We assessed correlation of covariates and ensured that the correlation coefficient between each covariate was below *ρ < 0.7*. We also inspected variance inflation factors (calculated using the car package v3.1–2) when making choices between correlated variables. Although the variable of annual summer precipitation has been suggested to be important to the distribution of the Gila monster (Lovich and Beaman 2007), it was not considered due to high correlation (*p > 0.8*) with, and higher VIF (10.3 vs. 6.4), compared to annual winter precipitation.

**TABLE 1:** Environmental covariates used to model suitable habitat for Gila monsters in the Mojave desert using ensemble modeling. All layers were scaled to a 250 m resolution for habitat modeling.

Name	Description	Source	Reason
Coarse fragments	Volumetric measure of the amount of larger (> 2 mm, < 25 mm) soil particles	Soil Grids 250 m project (Hengl et al. 2017).	Soil characteristics are important in determining suitable shelter substrate and vegetation communities.
Surface Texture	Measure of the texture of the ground surface. Derived from ASTER and MODIS satellite imagery.	(Nowicki et al. 2019)	Surface texture is important in determining thermal environments of shelter sites.
Mountain Bases	30 m cells that (1) had less than 10° slope, (2) had a profile curvature value between −6.0 × 10^−4^ and − 1.8 × 10^−4^, and (3) surface roughness between 0.7 × 10^−8^ and 5.8 × 10^−8^.	Originally derived for (Inman et al. 2014)	Mountain bases offer more microclimate variability which may be important for thermoregulation.
Depth to Bedrock	Distance between the soil surface and bedrock.	Soil Grids 250 m project (Hengl et al. 2017).	The depth to bedrock can affect the availability of shelters for Gila monsters.
% Sand	An estimate of areas with relatively little vegetation derived from NDVI layers.	Soil Grids 250 m project (Hengl et al. 2017).	The amount of sand in the soil is important in thermal properties of an area.
Topographic Index	A measure of whether an area is in a valley/ridge top.	Derived from USGS Digital Elevation Model.	Topographic index is an important consideration in habitat structure of an area.
% Washes	An estimate of the density of washes (dry stream beds) in an area.	Originally derived for (Inman et al. 2014)	Desert washes are important habitat characteristics in the Mojave that offer diverse microhabitats and microclimates.
Average Spring Temperature	Averaged temperature of March–May.	CMIP5 (Taylor et al. 2012)	Activity of Gila monsters in our study area is primarily limited to the spring months, thus temperatures would determine the window of activity (Beck 2005)
Winter Precipitation	Winter precipitation from November through March.	CMIP5 (Taylor et al. 2012)	Precipitation in the Mojave Desert primarily falls in the winter, and is important in annual productivity of the ecosystem (Beatley 1969)

As Gila monsters are infrequently encountered, even when they are actively searched for (> 400 person/h per observation, Gienger, *unpublished data*), many of the locality points are concentrated in easy‐to‐access areas (e.g., state parks, national conservation areas). To account for this sampling bias, we created a raster layer that approximated the amount of search effort to “sample” an area for reptiles. To do this, we downloaded locality points for all snakes and uncommon lizards (*Crotaphytus bicintores, Dipsosaurus dorsalis, Sauromalus ater, Gambelia wislizenii*) from the study area from the same databases that we used to source the Gila monster localities with the reasoning that if these species were reported, a Gila monster would also have been reported. All areas in our study area that have had focused surveys for Gila monsters have records for some of these species. Furthermore, the highway network was well represented by this layer; nocturnal road cruising has long been a popular reptile sampling technique in the southwestern US and occasionally results in Gila monster records (Rosen and Lowe 1994). We then created a bias raster using a kernel density estimator, using the default kernel bandwidth, with the function “sp.kde” in R package spatialEco v2.0–0 (Evans 2021). We used this bias raster to weight the Gila monster pseudo‐absence points used in the species distribution model. Similar methods have been shown to improve predictions of species distributions (Inman et al. 2021). For example, if a pseudo‐absence point fell in an area that had a high number of reports of other reptile species, the point would have a higher weight than a point that fell in an area with no reports of other species.

To further reduce potential bias in our predictions, we used two cross‐validation methods to fit and evaluate all habitat models. In this process, each algorithm was fit across 20 iterations of randomly selected, spatially thinned presence points, with a 20% random sample (without replacement) withheld for model evaluation (blind) at each iteration (i.e., 80% of presence points were used in model training, and 20% in model testing). Pseudo‐absence points were also randomly drawn for each cross‐validation iteration. Further, model importance and performance scores were also calculated using 10 iterations of an 80/20 random selection of training and testing data.

Metrics of prediction accuracy of the model were calculated based on the evaluation data for each of the cross‐validation runs, and these metrics were subsequently averaged across runs for final models of individual algorithms and the final ensemble. Performance metrics included several threshold‐independent measures: AUC (the area under the receiver operating characteristic curve; (Fielding and Bell 1997)), the Boyce Index (BI; (Boyce et al. 2002; Hirzel et al. 2006)), and the True Skill Statistic (TSS; Allouche et al. 2006). The TSS considers both omission and commission errors (Allouche et al. 2006). TSS can be sensitive to prevalence, especially when quality absence data are not available, so we additionally assessed our final model with the Sorenson's similarity index (Leroy et al. 2018). We set a threshold for our final ensemble model using the maximum of summed sensitivity and specificity, and then calculated Sorenson's index using the equation in (Leroy et al. 2018).

Habitat distribution models vary in their ability to effectively discriminate between different classes of habitat along the full range of habitat suitability values (0–1; Hirzel et al. 2006). To evaluate this property of our model predictions, we calculated the continuous Predicted/Expected ratio curves for different point densities based on the BI (Hirzel et al. 2006) using the ecospat package (v3.1; Di Cola et al. 2017) in R. These curves reflect how well each model deviates from random expectation and inform the interpretation of habitat suitability categories by indicating the effective resolution of suitability scores for each model (i.e., the model's ability to distinguish different classes of suitability; Hirzel et al. 2006).

To generate predictive layers of habitat suitability, we selected the top candidate models from each algorithm, based upon model performance metrics across cross‐validation runs where models above the 50th quantile of AUC scores were selected for model averaging and prediction. Ensemble predictions for individual algorithms were generated by taking the weighted average of the candidate models for each algorithm type (i.e., the higher performing models for the RF, GBM, etc. algorithms), where the weights determined by TSS scores for each of the contributing models. Layers representing the standard error of the overall ensemble habitat suitability model were calculated as the standard deviation in model predictions across all candidate models, divided by the square root of the number of candidate models considered. The same approach was used to derive layers of standard deviation within each individual algorithm type.

### 2.3 Future Habitat Suitability Predictions

Model predictions of future climates were obtained by downscaling monthly forecasts for the global habitat for two different RCP (representative concentration pathways) scenarios from the CMIP5 output of the NCAR CCSM4 GCMs, which were predicted from 2012 to 2099 (Taylor et al. 2012). This GCM has five realizations that are run with relatively variable output, and we averaged across them to provide a more stable representation of the model outputs for each scenario. GSMs from CCSM4 have been shown to represent a relatively unbiased prediction that trends toward the average of the CMIP5 models for the southwestern US, and thus were deemed appropriate for this research effort (Lee et al. 2019; Zobel et al. 2018).

The scenarios presented here are RCP 2.6 and RCP 8.5—which represent extremes in future climate scenarios to explore the potential magnitude of the differences in future suitability. RCP 2.6 represents a pathway in which carbon dioxide emissions are eliminated by 2100 and RCP 8.5 represents a pathway in which carbon dioxide emissions continuing to rise throughout the century (Taylor et al. 2012). The original data are provided at resolutions of (~1°x1° grid‐cell resolution or ~ 111 km; (Gent et al. 2011)), and they were downscaled using the delta method (Gleick 1986) to an 800 m x 800 m grid using PRISM climate data as the reference (PRISM Climate Group, Oregon State University 2022). For the purposes of this modeling effort, the data were then resampled to the 250 m x 250 m grid used for modeling using a cubic spline method (project function in the terra package for R; v1.7–18 [Hijmans 2023]).

The SDMs produced use a 30‐year climatology averages for each of the climate parameters, which integrates over time, and it models the environment assuming stability with respect to climate. Because the monthly climate predictions contain substantial variability (especially for precipitation), single‐year habitat predictions were more erratic than we thought reasonable. We used 10‐year averages for the variables of interest (winter precipitation, average spring temperature) for each decade until the CMIP5 projection ends at the decade beginning in 2082.

### 2.4 Movement Model

The goal of this model was to understand environmental features that influence Gila monster movement decisions and to predict areas that may be unsuitable for Gila monster dispersal. We leveraged three Gila monster telemetry datasets collected in Clark County to relate movements of animals to environmental covariates. The site A dataset was collected 2001–2004 and included 12 individuals, site B 2013–2017 for 17 individuals, and the site C dataset was collected 2016–2021 and included 33 individuals. The datasets from sites A and C were collected on at least a bi‐daily basis during the primary active season of Gila monsters in the Mojave Desert (spring‐early summer, Beck 2005), the dataset for site B was collected on roughly a weekly basis. All sites consisted of varied, often rocky terrain with Mojave Desert scrub vegetation associations (Turner 1994). Site A was roughly 650 m in elevation, Site B roughly 900 m in elevation, and Site C was roughly 1200 m in elevation; sites were generally characteristic of where most Gila monster records are known from the Mojave Desert. Telemetry data from animals with sufficient data for analysis (> 1 year of data and > 40 telemetry locations; *n* = 35 individuals) were thinned by removing consecutive relocations where the animals did not move, relocations taken longer than 36 h from prior locations, and relocations fewer than 50 m apart, leaving a dataset that only represents actual movements.

Similar to methods used to model Mojave desert tortoise ( *Gopherus agassizii* ) movement in Gray et al. (2019) and developed in McClure et al. (2017), we fit Brownian Bridge movement models (BBMMs) to the telemetry data from sites A and C to derive an empirical estimate of Gila monster movements via determination of occurrence probabilities (Horne et al. 2007). Brownian bridges were fit for each animal using the package BBMM (v3.0, Nielson et al. 2015) in program R (version 4.0.4, R Core Team 2018) and output as a raster which represented a probability surface of where each Gila monster moved. We then sampled 100 random points over each Brownian bridge raster, with a cutoff value of the model surface at 0.00000001 movement probability to sample over areas that each animal could have moved through, but did not, during our study period.

We considered several different environmental variables that we believed would influence Gila monster movement. Gila monsters inhabit areas with rugged terrain, and we anticipated that topographical features would influence movement decisions. We tested three different topological variables: slope (derived from the USGS digital elevation model (U.S. Geological Survey 2017)), TPI (topographic position index derived from the USGS digital elevation model), and a measure of topographic roughness (Dilts et al. 2023; Sappington et al. 2007). We also used the percent shrub cover dataset from the National Land Cover Dataset (NLCD) for 2003 for Site A and 2018 for Site C (Rigge et al. 2021). Shrub cover can be an important feature in desert landscapes, especially to ectothermic organisms that need shade resources to behaviorally thermoregulate (Grimm‐Seyfarth et al. 2017; Snyder et al. 2019). Finally, to account for anthropogenic alterations to both landscapes, we used a distance‐to‐feature layer for both paved roads and recreational hiking trails. Shapefiles for roads and paths from both sites were sourced from OpenStreetMaps (OpenStreetMap contributors 2021). All raster layers for movement model covariates were used at their original resolution of 30 m.

We extracted environmental values from environmental rasters for each random point within each BBMM raster. To relate the probability of movement to the environmental values, we fit linear mixed effects models in the package lme4 in R (v1.1–30; Bates et al. 2015). We log‐transformed the response variable to account for nonnormality in the modeled residuals. We used a random intercept for each individual animal to account for differences in sampling, movement patterns, and habitat availability. We considered all natural covariates along with their quadratic terms. For the distance‐to‐feature covariates (road/paths), we used a log‐distance relationship as responses to these localized covariates are not expected to be linear. Significance testing was done using Satterthwaite's method in package lmerTest and alpha = 0.05 (v3.1–3; Kuznetsova et al. 2017). No environmental covariate correlated with another with a coefficient greater than *ρ* = 0.4, so all were retained. We used the data from the site B study area to validate the model by assessing the predicted movement raster values at telemetry points.

### 2.5 Future Occupiable Habitat Projections

We expected that dispersal capability would be a limit to the occupation of future suitable habitat for Gila monsters. Dispersal has not been noted in previous telemetry studies of the species (Beck 1990, 2005; Beck and Jennings 2003; Gienger 2003; Kwiatkowski et al. 2008). One of the animals we tracked at Site C made a roughly three‐kilometer movement from its original capture location and never returned to the area where it was originally captured in over the next three years of tracking (Stalker et al. 2023). The individual was one of the smaller individuals in the study; this movement is best interpreted as a dispersal movement.

For each decadal habitat projection, we took the raster representing newly suitable habitat, created with a threshold that maximized the sum of sensitivity and specificity (Liu et al. 2005). We then masked out pixels that either fell more than 3 km from the edge of prior suitable habitat or overlapped with the 1st percentile of the movement model prediction at locality points used for the SDM, thus restricting our projections to areas that Gila monsters could disperse into and inhabit. We also masked out pixels with greater than 20% impervious surface from the NLCD % impervious layer (Homer and Fry 2012) to remove any area subject to extensive human development. Although Gila monsters can persist within areas with low levels of urbanization (Smith et al. 2010), highly developed areas with paved roads pose a high risk to slow moving Gila monsters, and developed areas reduce the length of movements made by resident Gila monsters (Kwiatkowski et al. 2008), thus they would likely restrict movements made by dispersing animals as well.

### 2.6 Gap Analysis

We wanted to understand how well the current protected area network within the study area encompasses both predicted current and future Gila monster habitat. We used a Gap Analysis, which is intended to estimate how much of a biological resource (e.g., a species' range) falls within areas that are considered protected from human development (Scott et al. 1993). We used the Protected Areas Database (PAD) of the U.S. which contains information on the protected areas within the U.S. and their management agencies (U.S. Geological Survey (USGS) Gap Analysis Project (GAP) 2022). We assessed how much and what proportion of Gila monster habitat above our determined threshold fell within the “Proclamation” and the “Designated” shapefiles from the PAD: “Proclamation” represents areas that have been set aside by U.S. legislation (e.g., National Parks, National Wildlife Refuges), while “Designated” areas are determined by acts of the executive branch (e.g., National Monuments, Areas of Critical Environmental Concern, Wilderness Areas). These different types of areas have different levels of permanence as “Designated” areas can be reclassified by different administrations, while “Proclamation” areas would require legislative action. We made the following modifications. First, we removed lands managed by tribal governments and the Department of Defense from the “Proclamation” category. Although these lands may contain suitable habitat, they also have usage needs that are unknown or can be incompatible with biodiversity conservation (e.g., Heaton et al. 2008). Second, to ensure areas were not counted twice, we did not consider “Designated” areas contained within “Proclamation” areas (e.g., a Wilderness Area inside a National Park) as separate from the “Proclamation” area. Third, we included areas in the “Fee” layer that were owned by state government agencies and nongovernmental organizations that were devoted to land preservation (e.g., state parks and private preserves) as part of the “Proclamation” layer. Fourth, as a separate layer, we also determined how much area fell within Bureau of Land Management (BLM) lands that are managed for multiple uses to understand how much suitable habitat falls within public lands that could potentially be placed under higher conservation status. Solar energy development has become an important land use of public land in the southwestern United States and often results in the permanent alteration of wildlife habitat (Karban et al. 2024). To assess if current and future habitat for Gila monsters has been designated for utility‐scale solar energy development we assess how much and what proportion of Gila monster habitat falls within BLM land designated “Available” in the Proposed Western Solar Plan (Bureau of Land Management 2024).

## 3 Results

**FIGURE 2:**
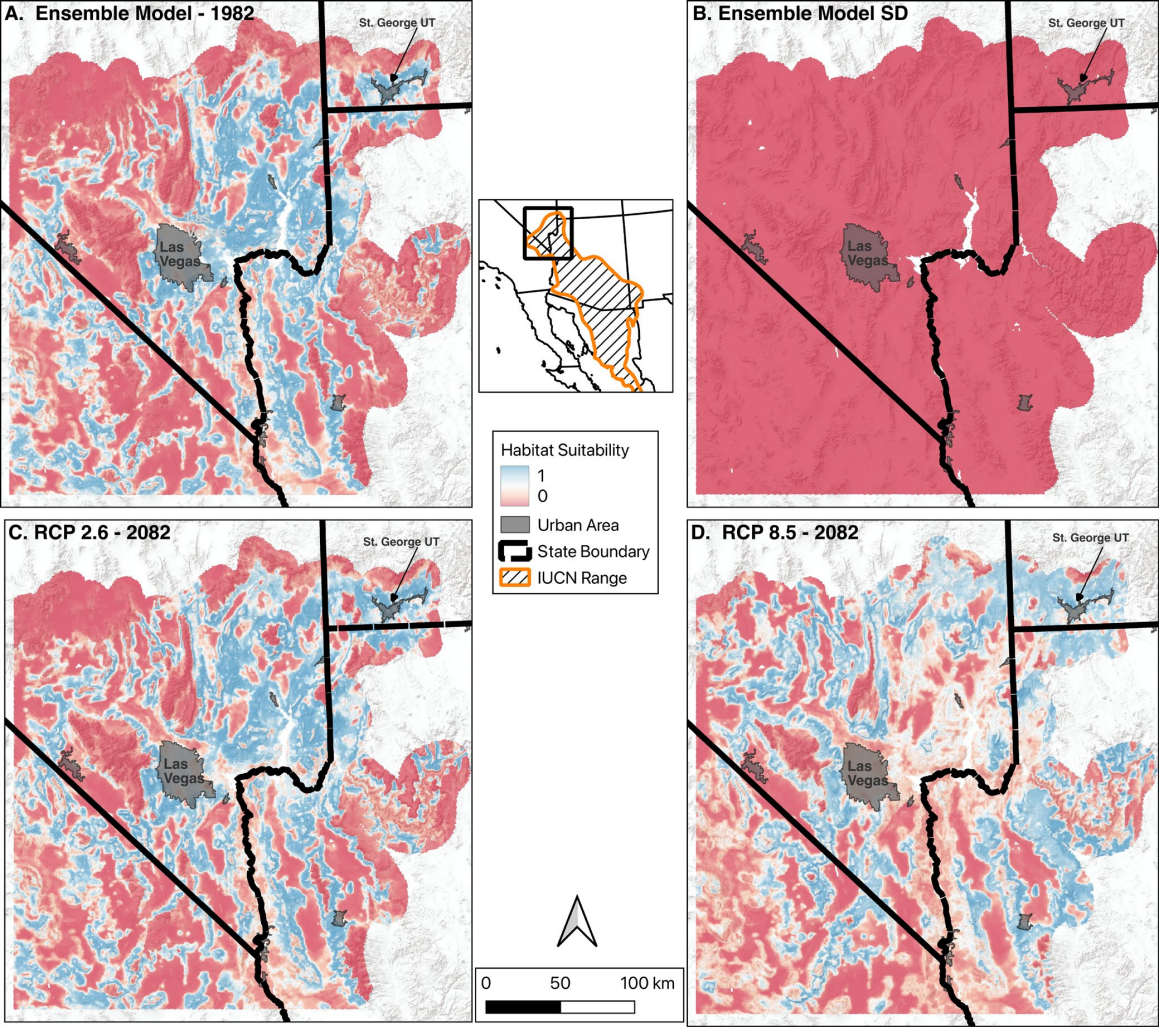
Results from an ensemble species distribution model and projections in future climatic conditions for the Gila monster in the Mojave Desert study area. Panels A and B show predicted suitability and standard deviation for historic (climatic averages of 1982) predicted Gila monster habitat. Standard deviation was low for the modeled area. Panels C and D show projected suitable habitat for Gila monsters under the RCP 2.6 and RCP 8.5 scenarios for the 2082–2091 period in the Mojave Desert study area.

The individual models predicting current distribution of Gila monsters in our study area performed well in all metrics (AUC, BI, TSS; Table 2), and the ensemble model had higher performance than any of the constituent individual model algorithms. After thresholding our ensemble model at the maximum of summed sensitivity and specificity at the modeled value of 0.49, our calculated Sorenson's index was 0.95, indicating that our model predicts observations well despite unknown true prevalence across the study area (Leroy et al. 2018). The continuous Boyce Index indicates excellent performance, with high discrimination ability across the entire prediction range. Standard error of the ensemble model indicates relatively low error rates overall ranging from 0 to 0.02 (Figure 2).

**TABLE 2:** Model diagnostics for individual models and the ensemble model used to determine historic habitat suitability for Gila monsters in the Mojave Desert study area using the testing dataset. AUC is Area under the Curve, BI is Boyce's Index, and TSS is true skill statistic. Sorenson similarity index was only calculated for the final ensemble model.

Model	AUC	BI	TSS	Sorenson
Ensemble	0.96	0.96	0.81	0.95
GAM	0.94	0.91	0.77	
RF	0.97	0.92	0.81	
Maxent	0.94	0.98	0.77	
GBM	0.94	0.89	0.79	

Our predictions of most current suitable habitat for Gila monsters fall within moderately rugged areas of Mojave Desert scrub vegetation. Large patches of suitable habitat occur between the Las Vegas Valley, Nevada, and the area around St. George, Utah, while the species appears to be limited mainly to the lower elevations of mountain ranges in the southern portion of the study area, and absent from areas of flat desert scrub. Projected suitable habitat for the RCP 2.6 and 8.5 scenarios in 2082 predicts increases in higher elevations of mountain ranges (Figure 2). Projected suitability for the RCP 2.6 scenario in the lower elevations of currently suitable habitat is predicted mostly to be the same in 2082 but is greatly reduced in the RCP 8.5 scenario.

### 3.1 Movement Model

Our results supported a nonlinear (quadratic) relationship of movement probability with slope, shrub cover and ruggedness, and a linear relationship with TPI. We found a negative relationship between Gila monster movement probability and topographic position, a positive relationship with areas of moderate slope, higher shrub cover, and closer to roads/hiking trails (Table 3). All covariates were significantly different from zero except for the linear term for terrain ruggedness.

**TABLE 3:** Relationship between probability of Gila monster movement and covariates for movement model. Distance to roads and path were fit after performing a log‐transform to the covariate.

Covariate	Linear term	Quadratic term	*p*
Slope	+	−	< 0.001, < 0.001
Ruggedness	+	−	0.11, 0.01
Topographic Position Index	−		< 0.001
Shrub Cover	+	−	< 0.001, < 0.001
Habitat Suitability	+		< 0.001
Distance to Road	−		< 0.001
Distance to Path	−		< 0.001

**FIGURE 3:**
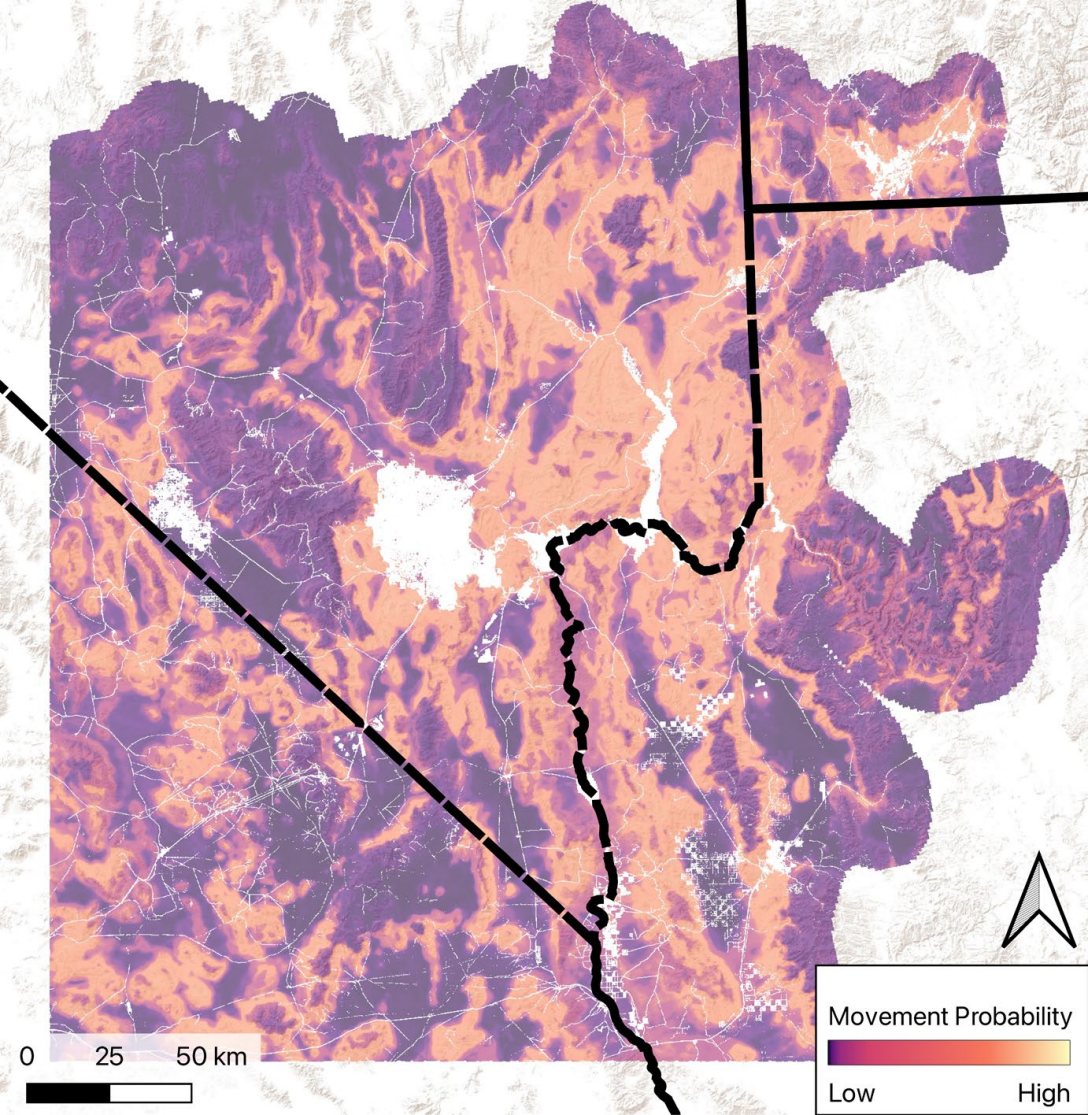
Map showing movement probability for Gila monsters in the Mojave Desert study area. Light values indicate areas that have a high probability of Gila monster movement. White areas indicate areas masked out due to high impervious surface cover or open water, as these represent likely barriers to Gila monster movement.

Our predictions over the study areas (Figure 3) indicate that Gila monster movement is likely to be greatest in sloped terrain, but not in areas of extreme slopes. Areas of flat terrain with lower vegetation cover (typically creosote flats in valley bottoms) have poor movement potential, though some deeply incised washes offer high quality habitat for movement through some areas. Values of the movement model prediction for telemetry points at Site B were above the 80th percentile of all predicted pixels indicating good predictive power.

### 3.2 Future Occupied Habitat Projections

**FIGURE 4:**
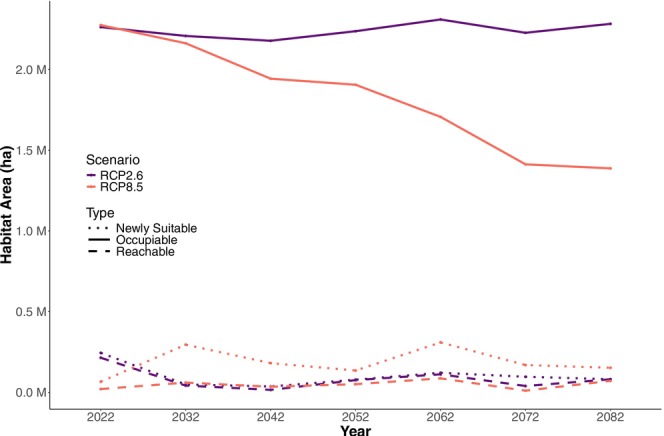
Projections of amount of habitat (in hectares) for Gila monsters in the Mojave Desert over time in two different emission scenarios (colors). Occupiable habitat (solid lines) includes formerly suitable habitat that remains suitable and newly suitable habitat that could be reached by dispersing Gila monsters. Newly suitable habitat (dotted lines) is habitat modeled as newly suitable under new conditions. Reachable habitat (dashed lines) is newly suitable habitat likely colonizable by dispersing Gila monsters.

**FIGURE 5:**
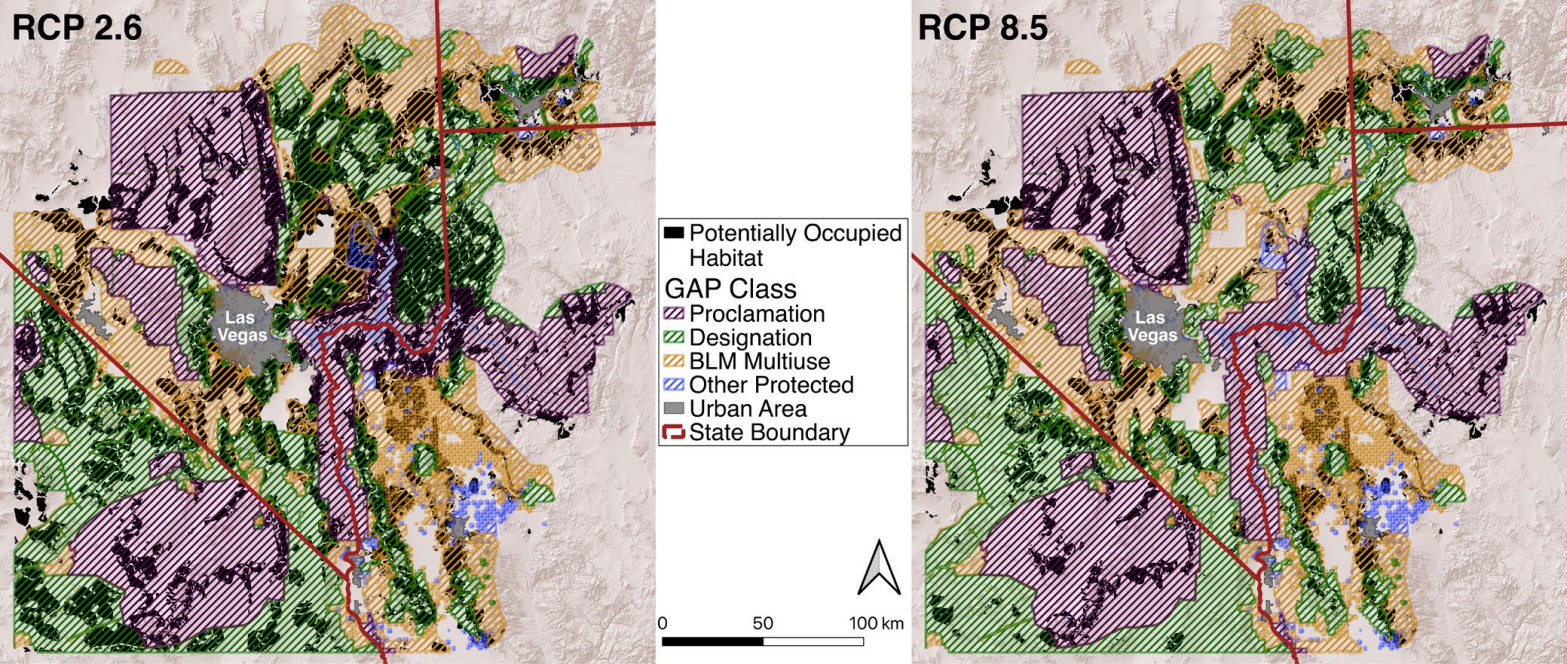
Future suitable and reachable habitat (black areas) for Gila monsters in the study area under both climate projections in 2082–2091 overlain on different land conservation designations (colored hashed areas).

The area of potentially occupiable habitat for Gila monsters within the study area by the decade beginning in 2082 under the RCP 2.6 emissions scenarios increases to 104% of currently suitable habitat and decreases to 63% of current suitable habitat under the RCP 8.5 scenario (Figure 4). Most of the remaining potentially occupiable habitat in both scenarios is in the area around the edges of the larger mountain ranges, and in the northeastern portion of the study area (Figure 5).

The RCP 8.5 climate projection indicated less habitat would be potentially occupied in lower elevation areas around mountain ranges and indicated that large areas of formerly suitable habitat in the areas northeast of the Las Vegas metro area would be lost as Gila monster habitat (Figure 5). Our results suggest that areas in the lower elevations of the Spring, Sheep, Muddy, McCullough, Virgin, and Mormon Mountains may maintain suitable habitat for Gila monsters under the RCP 8.5 scenario.

### 3.3 GAP Analysis

Current and future modeled Gila monster habitat falls almost entirely (> 90%) within public land in our study area, and the proportion of habitat within protected areas remains stable (roughly 60%) across the different emissions scenarios (Table 4). About twice as much suitable habitat is predicted to fall within “Designation” areas protected by executive branch action than “Proclamation” areas protected by management plans from land management agencies (~40% vs. ~20%), though this ratio and total area is smaller in the RCP 8.5 projection. The proportion of suitable habitat in other protected areas remains small across both projections. Only small portions (3% or less) of current and future Gila monster habitat falls within BLM land designated for utility‐scale solar development (Table 4).

**TABLE 4:** Proportion (and total area in ha) of suitable habitat for Gila monsters that falls within different GAP classifications across different emission scenarios. “Proclamation” areas are set aside by legislative action, “Designation” by agency action, “Other” by state and local action. “BLM” incorporates all BLM land open to multiple use, and “Solar” incorporates all BLM land that is proposed to be available for utility scale solar development.

	Proclamation	Designation	Other	BLM	Total protected	Total public	Solar
Current	0.20 (437 k ha)	0.43 (920 k ha)	0.01 (31 k ha)	0.25 (541 k ha)	0.65 (1.39 M ha)	0.90 (1.93 M ha)	0.03 (89 k ha)
2082 (RCP 2.6)	0.22 (497 k ha)	0.43 (897 k ha)	0.01 (28 k ha)	0.26 (603 k ha)	0.66 (1.42 M ha)	0.92 (2.0 M ha)	0.03 (59 k ha)
2082 (RCP 8.5)	0.24 (332 k ha)	0.36 (505 k ha)	< 0.01 (13 k ha)	0.30 (423 k ha)	0.61 (.85 M ha)	0.91 (1.3 M ha)	0.02 (32 k ha)

## 4 Discussion

We determined the distribution of current habitat in the Mojave Desert for the Gila monster and projected the likely distributions for different climatic futures. Our results indicate knowledge of dispersal capabilities with suitable habitat modeling leads to more restricted and realistic predictions of occupied habitat. Without this dispersal restriction, a much larger area would be projected as suitable for the species although occupancy would likely not occur in these areas. Thus, even though our knowledge of Gila monster dispersal ecology is limited, incorporating these limitations are important predictors of the future for this iconic species under differing climatic scenarios.

Our results show a starkly different projections of future range of the species between the two emissions scenarios examined here. Projections of habitable area in the low emissions scenario are predicted to remain relatively constant; not much habitat that is currently suitable would likely be lost, and newly suitable habitat appears in proximity to suitable habitat (Figure 4). In contrast, the high emissions scenario is predicted to result in a much‐reduced area of suitable habitat that has the potential for Gila monsters (Figure 4). In this more dire scenario, new areas of potentially suitable habitat are often located too far from areas that have potential occupancy and are never able to be occupied via natural dispersal. These contrasting results provide further weight to the importance of reducing carbon emissions to protecting native biodiversity (Pecl et al. 2017). If emissions are not controlled, Gila monster habitat is predicted to become highly fragmented (Figure 5), which may pose extinction risks for the species due to the loss of genetic and demographic connectivity (Saunders et al. 1991; Vandergast et al. 2016). Predicted habitat fragmentation is especially high in the RCP 8.5 scenario; most lower elevation habitat will become unsuitable, and remaining patches in lower elevation mountain ranges (e.g., the Muddy Mountains, Newberry Mountains) are predicted to be smaller and more isolated than in the RCP 2.6 scenario (Figure 5). It is unknown how large a patch of habitat must be to support a population of Gila monsters, though populations in smaller patches could be more sensitive to stochastic effects and genetic isolation from other populations (Vandergast et al. 2016).

Dispersal, a key process in colonizing new habitat, has not been noted in previous telemetry studies of the species (Beck 1990, 2005; Beck and Jennings 2003; Kwiatkowski et al. 2008). Adult Gila monsters are highly philopatric (Stalker et al. 2023), and individuals that were experimentally translocated were found to move up to a kilometer to return to their home range (Sullivan et al. 2004). This lack of dispersal information may be due to limitations on telemetry studies; all studies have assessed movements of adult Gila monsters due to constraints on implantation of radios and difficulties in finding juvenile individuals. Dispersal in lizards often occurs in the juvenile life stage (Sinervo et al. 2006; Templeton et al. 2011), and studies of juvenile Gila monster movements have not been conducted to our knowledge. There is only one known example of Gila monster dispersal; this apparent subadult individual crossed an area of currently suitable habitat during its ~3 km dispersal event (Stalker et al. 2023). It is unknown if dispersing Gila monsters make different movement choices from nondispersing animals—dispersing animals may be more likely to cross areas that are not considered suitable and typical dispersal movements may be shorter or longer than 3 km. Dispersal propensity by other lizards (Massot et al. 2008) and the Mojave desert tortoise (Hromada 2022) have been linked to annual weather conditions. If dispersal propensity in Gila monsters changes under new climatic conditions, then genetic exchange between these newly isolated populations may be reduced further increasing the risk of genetic drift, especially under the high emissions scenario.

Behavioral modification might be important in mitigating effects of decreased climatic suitability for ectotherms; especially increases in temperature (R. Kearney 2013). Prior studies that did not account for behavioral thermoregulation predicted the near extinction of the entire Helodermatidae by 2080 (Sinervo et al. 2010), a prediction that is unsupported by our results. This discordance in predictions is likely due to (Sinervo et al. 2010) not accounting for behavioral thermoregulation and microclimatic variability (R. Kearney 2013). Gila monsters are flexible in their activity patterns in response to environmental conditions and resource availability, spending a majority of their time within shelters that buffer extreme temperatures; especially during hot and dry conditions (Beck and Jennings 2003; Davis and DeNardo 2010; Gienger et al. 2014). Depending on conditions, Gila monster activity can shift between diurnal and nocturnal periods (Beck 2005), and they can remain in hibernacula dens through the spring breeding season during extreme drought conditions (Hughes et al. 2021). Behavioral modifications may not fully mitigate against changes in the thermal environment (Díaz et al. 2022), yet the range of the species extends into the much hotter Sonoran Desert; studies on potential local adaptations of Gila monsters at range edges could provide important information on the potential adaptive capacity of the species under new climatic regimes (Aguirre‐Liguori et al. 2021; Nadeau and Urban 2019). Expanding modeling efforts to include the entire range, genetic structure, and energy budgets of the species would also be prudent to understanding the tolerances of the species, how interaction of climatic variables such as summer precipitation and temperature, and the adaptive potential of different populations.

We found that Gila monster movement is constrained by areas of low vegetative cover and extremely rugged topography. Although Gila monsters inhabit environments that experience extreme temperatures, they are sensitive to water loss and restrict activity above relatively low temperatures compared to many desert lizards (Beck 1990; Davis and DeNardo 2010). Gila monsters may choose to move through areas that offer protection (shelter sites, perennial vegetative cover) from environmental and other hazards, as has been suggested in other studies (Smith et al. 2010). Dispersal of a similarly‐sized lizard with likely similar movement capacity ( *Varanus varius* ) has been suggested to be limited by rugged topography (Smissen et al. 2013). Most newly suitable habitat was predicted to occur in areas that Gila monsters prefer to move through (moderately rugged with high vegetative cover), so restriction in potentially occupied habitat was not primarily due to movement propensity but dispersal distance. One factor that we could not well parameterize is how Gila monsters move at the edge of modeled suitable habitat. This may be important because if Gila monsters react to edges of what they perceive as suitable habitat by turning around they may not disperse into areas that are modeled as becoming suitable in the future. There are rare records and descriptions of Gila monsters in vegetation communities that typically were found to be unsuitable under our model (e.g., pinyon‐juniper woodland; Beck 2005), suggesting that these may currently serve as marginal habitat that may better support the species with a change in abiotic conditions. We did find that Gila monsters often moved near trails and paved roads, which likely provide little to no resources for the species, though may be in areas of high resource availability (e.g., riparian zones). We attribute this to the fact that many of the Gila monsters used in this study were captured along roads or hiking paths, thus we attribute the “attraction” to these features as reflective of where these animals were captured and inhabited. These results suggest that these features are not actively avoided by Gila monsters when located within otherwise acceptable movement habitat, and pose a mortality risk (e.g., vehicle strikes, illegal collecting).

Ideally, projections of a species distribution into future climatic scenarios would include process‐based methods that include information on population‐level processes (e.g., demography, dispersal, recruitment) that would allow for populations to expand into newly available areas (Briscoe et al. 2019; Elith and Leathwick 2009). However, these processes in Gila monster populations remain poorly understood and difficult to properly document, especially in the northern part of their range where surface activity is limited. Research into populations in Utah, Arizona, and New Mexico suggests that individuals reach sexual maturity in roughly three‐to‐four years and adult annual survival is above 70% (Beck 1990, 2005; Smith et al. 2010). However, aside from a population subsidized by the watering of a golf course (Smith et al. 2010), little information exists on key demographic rates (e.g., juvenile growth, survival, adult fecundity, population size) restricting potential demographic modeling. Future projections of a species distribution under different climatic scenarios are only as good as the climatic projections used to make them (Beaumont et al. 2008); our use of the averaged CMIP5 projections have implications for our modeled habitat. One potential issue is that the mean precipitation projections for our study area remain relatively stable while the variance increases, and our habitat suitability model was fit to precipitation averages. These averaged projections do not capture the current mega‐drought in the western United States intensified by human climate change; future precipitation regimes in the study area are uncertain though drought is likely to continue (Coats and Mankin 2016; Williams et al. 2022). There are also many GCMs available with which to model that provide different possible future predictions. While CMIP5 has been shown to provide a reasonable estimate tending toward the average of many of these models (Zobel et al. 2018; Lee et al. 2019), other models may also provide a plausible solutions, and while they may add insights toward the range of possible outcomes, those were not the focus of this study, where we chose to focus on movement and dispersal potential in a fixed set of plausible scenarios. The role that precipitation patterns may play in Gila monster demography is not well understood, and future (and current) drought events may negatively affect the species and reduce populations in areas that may otherwise be suitable habitat. Another issue in projecting species distribution models to new climates can be transferability issues when environmental variables for future projections fall outside the environmental space of the training data (Owens et al. 2013). This was only true for a small portion of our study area, the lower elevations near the Colorado River, where climate in the RCP 8.5 projection was projected to get warmer and drier than current conditions in our study area and was classified as worse (but not completely unsuitable) habitat for Gila monsters. Suitability projections should be treated as uncertain in this area, though future conditions in these areas are similar to current climatic conditions areas where Gila monsters are absent farther south along the river.

Biotic interactions are also likely important in determining the distribution of Gila monsters. As primarily nest predators, the persistence of Gila monsters depends on the production of nests by a variety of bird, mammal, and reptile species that all have different abiotic needs (reviewed in Beck 2005). This generalist nature of the species' diet may provide some protection from future climatic shifts in prey species, though future research on how climatic conditions may alter the productivity of prey species would help to better understand how future climate scenarios may alter available prey resources. For example, production of eggs by Mojave desert tortoises depends on both precipitation and spring temperature (Mitchell et al. 2021), and heteromyid rodent density is predicted by seasonal precipitation (Beatley 1969), though desert cottontail abundance does not seem to be regulated by seasonal precipitation (Lightfoot et al. 2011). A better understanding of how biotic interactions play a role in determining the current distribution of Gila monsters would serve to better predict how changes in these interactions may alter the future distribution of the species.

Our results suggest that almost all current and future suitable Gila monster habitat (> 90%) in our study area falls within public lands, and a majority (> 60%) falls within areas that have some level of protection (Table 4). However, over half of this protected habitat falls within areas that have protection granted only by executive branch action (e.g., National Monument declarations under the Antiquities Act, Areas of Critical Environmental Concern designated by BLM offices), which can be removed by later executive branch administrations. Additionally, over 25% of future potentially occupied habitat for Gila monsters in both emission scenarios are located within BLM lands that are managed for multiple uses. Most suitable Gila monster habitat in our study area does not overlap areas designated for solar development, partially due to other actions to protect habitat for protected species and partially due to creosote flats not being typically suitable for Gila monsters. It is not clear which management actions may benefit Gila monster populations; though permanent protection of areas that are predicted to remain potentially occupied by the species would help ensure the survival of the species. Assessing how strategies to conserve populations of other at‐risk taxa, such as the desert bighorn sheep or Mojave desert tortoise, may benefit protection of Gila monster and ultimately, could be beneficial to optimizing conservation resources. Areas that have been deemed important for tortoise population connectivity fall within areas that we predict will remain occupied Gila monster habitat (Hromada et al. 2020); future efforts should continue to determine other areas that can benefit multiple at‐risk species.

## Author Contributions

**Steven J. Hromada:** data curation (equal), formal analysis (lead), investigation (equal), methodology (equal), software (equal), visualization (lead), writing – original draft (lead), writing – review and editing (lead). **Jason L. Jones:** conceptualization (equal), funding acquisition (equal), investigation (equal), project administration (equal), resources (equal), supervision (equal), writing – review and editing (equal). **Jocelyn B. Stalker:** data curation (supporting), investigation (equal), writing – review and editing (equal). **Dustin A. Wood:** conceptualization (equal), funding acquisition (equal), writing – review and editing (equal). **Amy G. Vandergast:** conceptualization (equal), funding acquisition (equal), writing – review and editing (equal). **C. Richard Tracy:** investigation (supporting), project administration (supporting), supervision (supporting), writing – review and editing (supporting). **C. M. Gienger:** conceptualization (equal), funding acquisition (equal), investigation (equal), project administration (lead), supervision (lead), writing – review and editing (equal). **Kenneth E. Nussear:** conceptualization (equal), data curation (equal), formal analysis (equal), funding acquisition (equal), methodology (equal), project administration (equal), software (equal), supervision (lead), validation (equal), writing – original draft (supporting), writing – review and editing (equal).

## Conflicts of Interest

The authors declare no conflicts of interest.

## Data Availability

There is demand for Gila monsters in the illegal wildlife trade, and poaching continues to be a risk for the species in the Mojave Desert. Because of this, data used for these analyses are not being publicly released as they would provide detailed localities of sensitive locations such as shelter sites.
